# Tetra­kis(μ-3-aza­niumylbenzoato)-κ^3^ *O*:*O*,*O*′;κ^3^ *O*,*O*′:*O*;κ^4^ *O*:*O*′-bis­[triaqua­chloridolanthanum(III)] tetra­chloride dihydrate

**DOI:** 10.1107/S1600536810052864

**Published:** 2010-12-24

**Authors:** Meriem Benslimane, Hocine Merazig, Jean-Claude Daran

**Affiliations:** a Unité de Recherche de Chimie de l’Environnement et Moléculaire Structurale, Faculté des Sciences Exactes, Département de Chimie, Université Mentouri de Constantine, 25000 Constantine, Algeria; b Laboratoire de Chimie de Coordination, UPR-CNRS 8241, 05 route de Narbonne, 31077 Toulouse Cedex 4, France

## Abstract

The tiltle complex, [La_2_(C_7_H_7_NO_2_)_4_Cl_2_(H_2_O)_6_]Cl_4_·2H_2_O, is a centrosymmetric dimer formed by edge-sharing LaO_5_(H_2_O)_3_Cl polyhedra linked together by a carboxyl­ate ligand. The two La^III^ metal ions are linked by two bidentate bridging carboxyl­ate groups with a κ^2^ *O*:*O*′ coordination mode and two bidentate chelating bridging carboxyl­ate groups with a κ^3^ *O*:*O*,*O*′ coordination mode. The coordination sphere of lanthanum, completed by a terminal chloride and three water mol­ecules, adopts a distorted tricapped trigonal–prismatic arrangement. N—H⋯Cl, N—H⋯O and O—H_water_⋯Cl hydrogen bonds, and slipped π–π inter­actions between parallel benzene rings [centroid–centroid distance of 3.647 (3) Å] are observed in the structure. These combine to stabilize a three-dimensional network.

## Related literature

For potential applications of lanthanide complexes, see: Aime *et al.* (1998 ▶); Bao *et al.* (2007 ▶); Drew *et al.* (2000 ▶); Ishikawa *et al.* (2005 ▶); Liu *et al.* (2004 ▶). For lanthanide complexes with organic ligands, see: Cao *et al.* (2002 ▶); Wang *et al.* (2000 ▶); Lam *et al.* (2003 ▶); De Sa *et al.* (1998 ▶); Serra *et al.* (1998 ▶); Bassett *et al.* (2004 ▶); Galaup *et al.* (1999 ▶); Blasse *et al.* (1987 ▶); Prodi *et al.* (1998 ▶); Ramirez *et al.* (2001 ▶); Thuery *et al.* (2000 ▶); Bunzli & Ihringer (2002 ▶); Jones *et al.* (1997 ▶); Bardwell *et al.* (1997 ▶); Horrocks *et al.* (1997 ▶). For similar complexes, see: Qin *et al.* (2005 ▶, 2006 ▶); Xiong & Qi (2007 ▶); Song *et al.* (2005 ▶); Anna & Kaziol (1999 ▶). For the use of the SQUEEZE function of *PLATON*, see: Spek (2009 ▶).
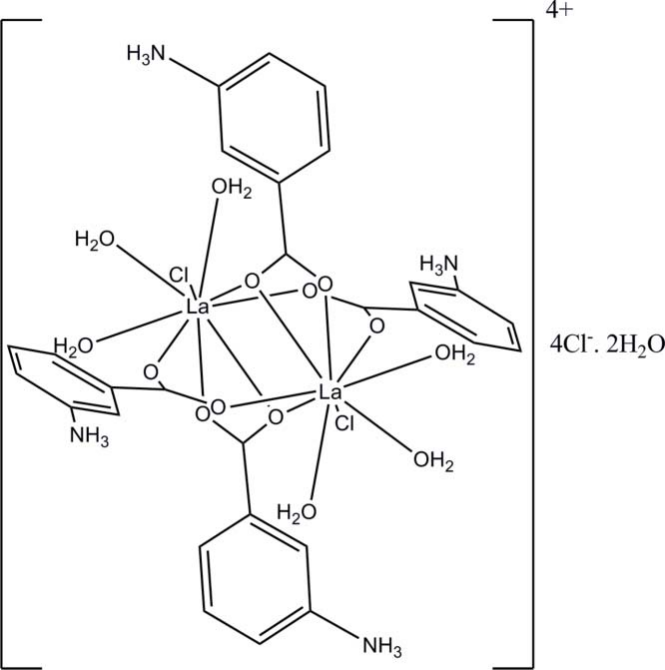


## Experimental

### 

#### Crystal data

[La_2_(C_7_H_7_NO_2_)_4_Cl_2_(H_2_O)_6_]Cl_4_·2H_2_O
*M* *_r_* = 1183.19
Monoclinic, 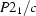

*a* = 11.2988 (3) Å
*b* = 19.8679 (4) Å
*c* = 10.4679 (3) Å
β = 112.693 (1)°
*V* = 2167.96 (10) Å^3^
*Z* = 2
Mo *K*α radiation
μ = 2.38 mm^−1^
*T* = 293 K
0.24 × 0.22 × 0.18 mm

#### Data collection

Enraf–Nonius CAD-4 diffractometer
Absorption correction: refined from Δ*F* (*DIFABS*; Walker & Stuart, 1983 ▶) *T*_min_ = 0.550, *T*_max_ = 0.789
6316 measured reflections
6315 independent reflections
4414 reflections with *I* > 2σ(*I*)
*R*_int_ = 0.027
2 standard reflections every 60 min  intensity decay: 3%

#### Refinement

*R*[*F* ^2^ > 2σ(*F* ^2^)] = 0.044
*wR*(*F* ^2^) = 0.110
*S* = 1.00
6315 reflections
246 parameters
H-atom parameters constrained
Δρ_max_ = 2.85 e Å^−3^
Δρ_min_ = −0.87 e Å^−3^

### 

Data collection: *CAD-4 EXPRESS* (Enraf–Nonius, 1994 ▶); cell refinement: *CAD-4 EXPRESS*; data reduction: *XCAD4* (Harms & Wocadlo, 1996 ▶); program(s) used to solve structure: *SIR92* (Altomare *et al.*, 1993 ▶); program(s) used to refine structure: *SHELXL97* (Sheldrick, 2008 ▶); molecular graphics: *ORTEP-3 for Windows* (Farrugia, 1997 ▶); software used to prepare material for publication: *SHELXL97*.

## Supplementary Material

Crystal structure: contains datablocks I, global. DOI: 10.1107/S1600536810052864/su2239sup1.cif

Structure factors: contains datablocks I. DOI: 10.1107/S1600536810052864/su2239Isup2.hkl

Additional supplementary materials:  crystallographic information; 3D view; checkCIF report

## Figures and Tables

**Table 1:** Hydrogen-bond geometry (Å, °)

*D*—H⋯*A*	*D*—H	H⋯*A*	*D*⋯*A*	*D*—H⋯*A*
N1—H1*A*⋯Cl3	0.89	2.30	3.170 (5)	167
N1—H1*B*⋯Cl2^i^	0.89	2.43	3.214 (5)	147
N2—H2*A*⋯O4^ii^	0.89	2.45	3.046 (5)	125
N2—H2*A*⋯Cl2	0.89	2.49	3.221 (4)	140
N2—H2*B*⋯Cl3^iii^	0.89	2.28	3.169 (4)	177
N2—H2*C*⋯Cl1^ii^	0.89	2.49	3.215 (4)	138
N2—H2*C*⋯Cl1^iv^	0.89	2.72	3.349 (5)	128
O1*W*—H11⋯Cl2^ii^	0.81	2.39	3.186 (4)	170
O1*W*—H21⋯Cl2^v^	0.87	2.38	3.196 (4)	157
O2*W*—H12⋯Cl3^vi^	0.87	2.26	3.123 (4)	172
O2*W*—H22⋯Cl3^vii^	0.84	2.47	3.276 (4)	160
O3*W*—H13⋯O1*W*	0.79	2.41	2.920 (5)	124
O3*W*—H13⋯Cl2^v^	0.79	2.53	3.156 (4)	137
O3*W*—H23⋯Cl3^vii^	0.90	2.17	3.069 (4)	172

Symmetry codes: (i) 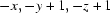; (ii) 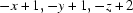; (iii) 
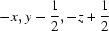
; (iv) 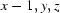; (v) 
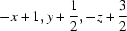
; (vi) 
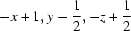
; (vii) 
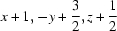
.

**Fig. 1.**
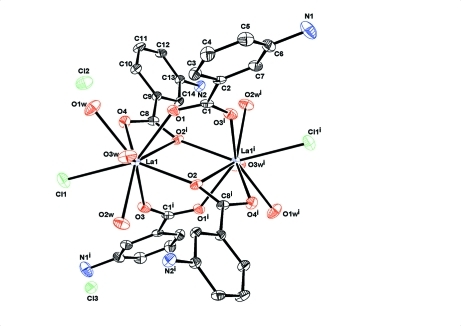
The molecular structure of the title compound, with the atom-numbering scheme. Displacement ellipsoids are drawn at the 30% probability level [Symmetry code: (i) -x + 1, -y + 1, -z + 1; Hydrogen atoms have been omitted for clarity].

**Fig. 2.**
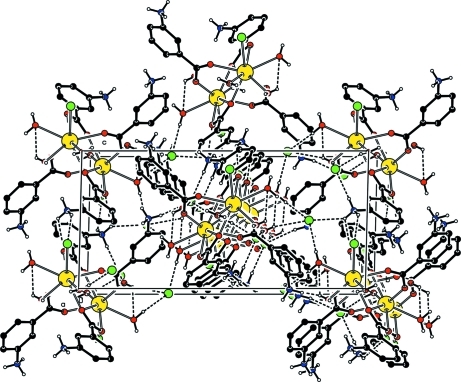
The crystal packing of the title compound, viewed roughly down the a axis. Hydrogen bonds are shown as dashed lines [see Table 1 for details; Hydrogen atoms not involved in hydrogen bonding have been omitted for clarity].

## supplementary crystallographic information

### Comment

The study of the coordination chemistry of lanthanide elements is a rapidly growing area of interest, as a result of potential applications of their complexes, as magnetic resonance imaging contrast agents (MRI) (Aime *et al.*, 1998), as catalysts in organic synthesis (Bao *et al.*, 2007), as molecular magnetic materials (Ishikawa *et al.*, 2005), in luminescence studies (Liu *et al.*, 2004) and in the solvent extraction of actinides (Drew *et al.*, 2000). In this field much work has been focused on the design and assembly of lanthanide complexes with organic ligands, such as carboxylic acids derivatives [Wang *et al.*, 2000; Cao *et al.*, 2002; Lam *et al.*, 2003;], β-dicetones [De Sa *et al.*, 1998; Serra *et al.*, 1998; Bassett *et al.*, 2004], cryptands [Galaup *et al.*, 1999; Blasse *et al.*, 1987], calixarenes [Prodi *et al.*, 1998; Ramirez *et al.*, 2001; Thuery *et al.*, 2000; Bunzli *et al.*, 2002], podands [Jones *et al.*, 1997; Bardwell *et al.*, 1997], heterocyclic ligands and proteins (Horrocks *et al.*, 1997). We report herein on the preparation and crystal structure of the title compound.

The molecular structure of the title compound consists of dimeric units related by an inversion centre (Fig. 1). Each La^III^ atom is nine-coordinated by five O atoms from carboxylate groups of the 3-ammoniumbenzoate, three O atoms from water molecules and one chloride anion. They adopt a distorted tricapped trigonal-prismatic arrangement. The two La^III^ atoms are linked by two bridging bidentate carboxylate groups and two bidentate chelating bridging carboxylate groups. A similar coordination environment was observed previously for lanthanoid(III) complexes, such as [Ln_2_(imidazole 4,5-dicarboxylate)_2_(H_2_O)_3_].1.5H_2_O (Ln = Sm and Eu; Qin *et al.*, 2005), [La_2_(pyridine-3,4-dicarboxylate)_2_(NO_3_)_2_ (H_2_O)_3_] (Qin *et al.*, 2006), and [La_2_(C_8_H_3_NO_6_)_2_(C_8_H_4_NO_6_)(H_2_O)_6_]_2_H_2_O (Xiong & Qi, 2007). The La···La distance is 4.2245 (5) Å, showing that there is no direct metal-metal bond between the La atoms. The La—O distances involving the carboxylate groups range from 2.453 (3) Å to 2.503 (3) Å, and those of the La-O_water_ bonds from 2.557 (3) Å to 2.618 (4) Å. All are within the range of those observed for other nine coordinate La^III^ complexes with oxygen-donor ligands (Song *et al.*, 2005; Anna & Kaziol, 1999). The carboxylate group shows a distortion from the molecular plane; the dihedral angle between the mean-planes of the benzene ring (C2-C7; plane 1) and the carboxlate group (O1/C1/O3) is 14.7 (6)°, and that between the mean-planes of benzene ring (C9-C14; plane 2) and the O2/C8/O4 carboxlate group is 24.6 (5)°. The two carboxylate groups are almost perpendicular to one another with a dihedral angle of 80.3 (8) °, and planes 1 and 2 are inclined to one another by 80.0 (2) °.

In the crystal hydrogen bonds involving the coordinated water molecules, the ammonium group NH_3_ and the Cl atom (free and coordinated) build up a three dimensionnal network (Fig. 2, Table 1). There are slipped π -π stacking interactions between the symetry (1 - *x*, 1 - *y*, 2 - *z*) related benzene rings (C9-C14) with a centroid-to-centroid distance of 3.647 (3) Å and an interplanar distance of 3.3607 (18) Å, leading to a slippage of 1.417 Å. Both hydrogen-bonding and π-π interactions combine to stabilize the three-dimensional network.

### Experimental

LaCl_3_.nH_2_O (0.25 g, 1 mmol) was dissolved in aqueous solution of NaOH (0.5*M*, 25 ml) with constant stirring. 3-aminobenzoic acid (0.11 g, 1 mmol) was added to the mixture and the pH was adjusted to ca. 3 using 4*M* HCl. The mixture was refluxed at 353 K for about 1 h and then cooled to room temperature. Slow evaporation of the solvent at room temperature lead to the formation of prismatic brown crystals of the title compound.

### Refinement

The unit cell contains some water molecules which appear to be highly disordered and it was difficult to model their positions and distribution reliably. The SQUEEZE function of *PLATON* (Spek, 2009) was used to eliminate the contribution of the electron density in the solvent region from the intensity data, and the solvent-free model was employed for the final refinement. There are four cavities of 27 Å^3^ per unit cell. *PLATON* estimated that the cavity contains 11 electrons which corresponds roughly to one water molecules per asymmetric unit or 2 water molecules per dimer. All H atoms attached to the aromatic C atoms were fixed geometrically and treated as riding with C—H = 0.93 Å and *U*_iso_(H) = 1.2*U*_eq_(C). The H-atoms of the coordinated water molecules and the amonium groups were located in difference Fourier maps and were initially refined using distance restraints (O—H and N—H = 0.85 (2) Å, and H···H= 1.40 (2) Å, with *U*_iso_(H) = 1.5*U*_eq_(O, N). However, in the last cycles of refinement, they were treated as riding on their parent atoms with AFIX 3 for the water H-atoms and AFIX 137 for the NH_3_ H-atoms (O-H = 0.79 - 0.90 Å; N-H = 0.89 Å). The highest peak in the difference map is 2.85Å located close to the La atom while the deepest hole is 0.87 Å.

### Crystal data

**Table:**

[La_2_(C_7_H_7_NO_2_)_4_Cl_2_(H_2_O)_6_]Cl_4_·2H_2_O	*F*(000) = 1168
*M**_r_* = 1183.19	*D*_x_ = 1.813 Mg m^−^^3^
Monoclinic, *P*2_1_/*c*	Mo *K*α radiation, λ = 0.71073 Å
Hall symbol: -P 2ybc	Cell parameters from 6316 reflections
*a* = 11.2988 (3) Å	θ = 1.0–30.0°
*b* = 19.8679 (4) Å	µ = 2.38 mm^−^^1^
*c* = 10.4679 (3) Å	*T* = 293 K
β = 112.693 (1)°	Prism, brown
*V* = 2167.96 (10) Å^3^	0.24 × 0.22 × 0.18 mm
*Z* = 2	

### Data collection

**Table:**

Enraf–Nonius CAD-4 diffractometer	*R*_int_ = 0.027
graphite	θ_max_ = 30.0°, θ_min_ = 2.0°
non–profiled ω/2τ scans	*h* = −15→14
Absorption correction: part of the refinement model (Δ*F*) *DIFABS* (Walker & Stuart, 1983)	*k* = 0→27
*T*_min_ = 0.550, *T*_max_ = 0.789	*l* = 0→14
6316 measured reflections	2 standard reflections every 60 min
6315 independent reflections	intensity decay: 3%
4414 reflections with *I* > 2σ(*I*)	

### Refinement

**Table:**

Refinement on *F*^2^	Primary atom site location: structure-invariant direct methods
Least-squares matrix: full	Secondary atom site location: difference Fourier map
*R*[*F*^2^ > 2σ(*F*^2^)] = 0.044	Hydrogen site location: inferred from neighbouring sites
*wR*(*F*^2^) = 0.110	H-atom parameters constrained
*S* = 1.00	*w* = 1/[σ^2^(*F*_o_^2^) + (0.060*P*)^2^] where *P* = (*F*_o_^2^ + 2*F*_c_^2^)/3
6315 reflections	(Δ/σ)_max_ = 0.001
246 parameters	Δρ_max_ = 2.85 e Å^−^^3^
0 restraints	Δρ_min_ = −0.87 e Å^−^^3^

### Special details

**Table:**

Geometry. All e.s.d.'s (except the e.s.d. in the dihedral angle between two l.s. planes) are estimated using the full covariance matrix. The cell e.s.d.'s are taken into account individually in the estimation of e.s.d.'s in distances, angles and torsion angles; correlations between e.s.d.'s in cell parameters are only used when they are defined by crystal symmetry. An approximate (isotropic) treatment of cell e.s.d.'s is used for estimating e.s.d.'s involving l.s. planes.
Refinement. Refinement of *F*^2^ against ALL reflections. The weighted *R*-factor *wR* and goodness of fit *S* are based on *F*^2^, conventional *R*-factors *R* are based on *F*, with *F* set to zero for negative *F*^2^. The threshold expression of *F*^2^ > σ(*F*^2^) is used only for calculating *R*-factors(gt) *etc*. and is not relevant to the choice of reflections for refinement. *R*-factors based on *F*^2^ are statistically about twice as large as those based on *F*, and *R*- factors based on ALL data will be even larger.

### Fractional atomic coordinates and isotropic or equivalent isotropic displacement parameters (Å^2^)

**Table:**

	*x*	*y*	*z*	*U*_iso_*/*U*_eq_	
C1	0.3737 (5)	0.6180 (2)	0.3817 (5)	0.0270 (9)	
C2	0.3278 (4)	0.6773 (2)	0.2831 (5)	0.0265 (9)	
C3	0.4148 (5)	0.7263 (2)	0.2812 (5)	0.0325 (10)	
H3	0.4985	0.7252	0.3468	0.039*	
C4	0.3781 (5)	0.7766 (2)	0.1827 (6)	0.0385 (12)	
H4	0.4376	0.8081	0.1791	0.046*	
C5	0.2527 (5)	0.7798 (3)	0.0900 (6)	0.0390 (12)	
H5	0.2264	0.8140	0.0243	0.047*	
C6	0.1667 (5)	0.7324 (3)	0.0949 (5)	0.0347 (11)	
C7	0.2016 (5)	0.6809 (2)	0.1899 (5)	0.0304 (10)	
H7	0.1417	0.6491	0.1914	0.036*	
C8	0.5026 (5)	0.5348 (2)	0.7463 (5)	0.0252 (9)	
C9	0.4230 (4)	0.5418 (2)	0.8325 (4)	0.0241 (9)	
C10	0.4425 (5)	0.5977 (2)	0.9175 (5)	0.0298 (10)	
H10	0.5041	0.6295	0.9208	0.036*	
C11	0.3714 (5)	0.6062 (2)	0.9963 (5)	0.0364 (11)	
H01	0.3814	0.6450	1.0491	0.044*	
C12	0.2846 (5)	0.5575 (2)	0.9982 (5)	0.0320 (10)	
H02	0.2373	0.5628	1.0532	0.038*	
C13	0.2694 (4)	0.5011 (2)	0.9176 (4)	0.0251 (9)	
C14	0.3360 (4)	0.4921 (2)	0.8324 (4)	0.0256 (9)	
H14	0.3231	0.4541	0.7767	0.031*	
N1	0.0341 (5)	0.7368 (3)	−0.0085 (6)	0.0559 (14)	
H1A	0.0143	0.7797	−0.0314	0.084*	
H1B	−0.0195	0.7199	0.0271	0.084*	
H1C	0.0274	0.7135	−0.0835	0.084*	
N2	0.1833 (4)	0.4481 (2)	0.9262 (4)	0.0328 (8)	
H2A	0.2289	0.4156	0.9820	0.049*	
H2B	0.1402	0.4312	0.8422	0.049*	
H2C	0.1282	0.4651	0.9597	0.049*	
O1	0.4840 (3)	0.62303 (16)	0.4762 (3)	0.0327 (7)	
O2	0.4623 (3)	0.49888 (16)	0.6384 (3)	0.0296 (7)	
O1W	0.7267 (4)	0.67276 (18)	0.6974 (4)	0.0509 (11)	
H11	0.7442	0.6831	0.7774	0.076*	
H21	0.7108	0.7058	0.6392	0.076*	
O3	0.3002 (3)	0.56857 (16)	0.3589 (4)	0.0361 (8)	
O2W	0.8388 (4)	0.5038 (2)	0.4974 (4)	0.0454 (9)	
H12	0.8859	0.4682	0.5280	0.068*	
H22	0.8907	0.5301	0.4821	0.068*	
O4	0.6072 (3)	0.56602 (17)	0.7855 (3)	0.0331 (8)	
O3W	0.7332 (4)	0.63595 (18)	0.4301 (4)	0.0459 (10)	
H13	0.7082	0.6667	0.4607	0.069*	
H23	0.8087	0.6298	0.4213	0.069*	
Cl1	0.92083 (12)	0.54510 (8)	0.82837 (14)	0.0468 (3)	
Cl2	0.24108 (15)	0.29052 (6)	0.99466 (15)	0.0445 (3)	
Cl3	−0.02500 (13)	0.88440 (7)	−0.13178 (14)	0.0400 (3)	
La1	0.67532 (2)	0.552876 (12)	0.58587 (2)	0.02160 (7)	

### Atomic displacement parameters (Å^2^)

**Table:**

	*U*^11^	*U*^22^	*U*^33^	*U*^12^	*U*^13^	*U*^23^
C1	0.036 (2)	0.022 (2)	0.030 (2)	0.0100 (18)	0.020 (2)	0.0042 (17)
C2	0.036 (2)	0.0151 (18)	0.033 (2)	0.0062 (17)	0.018 (2)	0.0008 (16)
C3	0.039 (3)	0.026 (2)	0.029 (2)	−0.002 (2)	0.010 (2)	0.0035 (19)
C4	0.044 (3)	0.028 (2)	0.045 (3)	−0.007 (2)	0.019 (2)	0.007 (2)
C5	0.053 (3)	0.029 (2)	0.039 (3)	0.006 (2)	0.022 (3)	0.015 (2)
C6	0.034 (3)	0.036 (3)	0.034 (2)	0.008 (2)	0.013 (2)	0.008 (2)
C7	0.036 (2)	0.024 (2)	0.038 (3)	0.0043 (18)	0.021 (2)	0.0068 (19)
C8	0.037 (2)	0.025 (2)	0.0213 (19)	0.0058 (17)	0.0186 (18)	0.0050 (16)
C9	0.027 (2)	0.026 (2)	0.0230 (19)	−0.0010 (16)	0.0134 (17)	−0.0017 (16)
C10	0.036 (3)	0.025 (2)	0.032 (2)	−0.0046 (19)	0.017 (2)	−0.0048 (18)
C11	0.050 (3)	0.026 (2)	0.042 (3)	−0.006 (2)	0.028 (2)	−0.012 (2)
C12	0.036 (2)	0.032 (2)	0.036 (2)	0.004 (2)	0.023 (2)	−0.004 (2)
C13	0.029 (2)	0.025 (2)	0.025 (2)	−0.0008 (17)	0.0142 (18)	0.0002 (17)
C14	0.031 (2)	0.026 (2)	0.022 (2)	−0.0022 (17)	0.0121 (18)	−0.0050 (16)
N1	0.040 (3)	0.053 (3)	0.066 (3)	0.010 (2)	0.011 (2)	0.029 (3)
N2	0.039 (2)	0.033 (2)	0.033 (2)	−0.0057 (19)	0.0202 (17)	−0.0008 (18)
O1	0.0361 (18)	0.0295 (17)	0.0296 (17)	0.0098 (14)	0.0094 (14)	0.0047 (14)
O2	0.0403 (19)	0.0284 (16)	0.0245 (15)	0.0020 (14)	0.0173 (14)	−0.0037 (13)
O1W	0.085 (3)	0.0282 (19)	0.035 (2)	−0.0070 (19)	0.018 (2)	−0.0013 (16)
O3	0.0395 (19)	0.0224 (16)	0.048 (2)	0.0059 (14)	0.0189 (17)	0.0113 (14)
O2W	0.044 (2)	0.049 (2)	0.053 (2)	0.0137 (18)	0.0309 (19)	0.0096 (19)
O4	0.0356 (18)	0.041 (2)	0.0288 (16)	−0.0065 (15)	0.0194 (14)	−0.0036 (14)
O3W	0.067 (3)	0.0277 (18)	0.065 (3)	−0.0033 (18)	0.050 (2)	0.0008 (17)
Cl1	0.0314 (6)	0.0699 (10)	0.0373 (6)	0.0061 (6)	0.0114 (5)	0.0087 (6)
Cl2	0.0609 (9)	0.0258 (6)	0.0508 (8)	−0.0008 (6)	0.0260 (7)	−0.0036 (5)
Cl3	0.0387 (7)	0.0364 (6)	0.0460 (7)	0.0029 (5)	0.0177 (6)	0.0077 (5)
La1	0.02571 (12)	0.02025 (11)	0.02189 (11)	0.00083 (11)	0.01252 (9)	0.00244 (11)

### Geometric parameters (Å, °)

**Table:**

C1—O3	1.249 (6)	C13—N2	1.460 (6)
C1—O1	1.261 (6)	C14—H14	0.9300
C1—C2	1.518 (6)	N1—H1A	0.8900
C2—C7	1.385 (7)	N1—H1B	0.8900
C2—C3	1.390 (6)	N1—H1C	0.8900
C3—C4	1.381 (7)	N2—H2A	0.8900
C3—H3	0.9300	N2—H2B	0.8900
C4—C5	1.376 (8)	N2—H2C	0.8900
C4—H4	0.9300	O1—La1	2.453 (3)
C5—C6	1.369 (7)	O2—La1^i^	2.484 (3)
C5—H5	0.9300	O2—La1	2.875 (3)
C6—C7	1.374 (6)	O1W—La1	2.618 (4)
C6—N1	1.474 (7)	O1W—H11	0.8086
C7—H7	0.9300	O1W—H21	0.8660
C8—O4	1.255 (6)	O3—La1^i^	2.472 (3)
C8—O2	1.263 (5)	O2W—La1	2.557 (3)
C8—C9	1.506 (6)	O2W—H12	0.8700
C8—La1	3.047 (4)	O2W—H22	0.8447
C9—C10	1.387 (6)	O4—La1	2.503 (3)
C9—C14	1.392 (6)	O3W—La1	2.575 (3)
C10—C11	1.367 (6)	O3W—H13	0.7901
C10—H10	0.9300	O3W—H23	0.9015
C11—C12	1.383 (7)	Cl1—La1	2.9545 (13)
C11—H01	0.9300	La1—O3^i^	2.472 (3)
C12—C13	1.372 (6)	La1—O2^i^	2.484 (3)
C12—H02	0.9300	La1—La1^i^	4.2245 (5)
C13—C14	1.383 (6)		
			
O3—C1—O1	126.5 (4)	C1—O3—La1^i^	135.8 (3)
O3—C1—C2	116.9 (4)	La1—O2W—H12	127.0
O1—C1—C2	116.6 (4)	La1—O2W—H22	119.0
C7—C2—C3	119.7 (4)	H12—O2W—H22	101.6
C7—C2—C1	120.5 (4)	C8—O4—La1	103.3 (3)
C3—C2—C1	119.7 (4)	La1—O3W—H13	91.2
C4—C3—C2	120.5 (5)	La1—O3W—H23	117.3
C4—C3—H3	119.7	H13—O3W—H23	130.3
C2—C3—H3	119.7	O1—La1—O3^i^	131.54 (12)
C5—C4—C3	119.4 (5)	O1—La1—O2^i^	71.07 (11)
C5—C4—H4	120.3	O3^i^—La1—O2^i^	77.85 (12)
C3—C4—H4	120.3	O1—La1—O4	80.34 (11)
C6—C5—C4	119.6 (5)	O3^i^—La1—O4	87.11 (12)
C6—C5—H5	120.2	O2^i^—La1—O4	123.36 (11)
C4—C5—H5	120.2	O1—La1—O2W	132.54 (12)
C5—C6—C7	122.0 (5)	O3^i^—La1—O2W	71.41 (12)
C5—C6—N1	117.8 (5)	O2^i^—La1—O2W	77.07 (12)
C7—C6—N1	120.1 (5)	O4—La1—O2W	147.12 (12)
C6—C7—C2	118.6 (5)	O1—La1—O3W	74.48 (12)
C6—C7—H7	120.7	O3^i^—La1—O3W	137.80 (12)
C2—C7—H7	120.7	O2^i^—La1—O3W	83.47 (12)
O4—C8—O2	122.7 (4)	O4—La1—O3W	134.14 (11)
O4—C8—C9	117.6 (4)	O2W—La1—O3W	67.65 (12)
O2—C8—C9	119.7 (4)	O1—La1—O1W	72.27 (13)
O4—C8—La1	53.1 (2)	O3^i^—La1—O1W	142.99 (13)
O2—C8—La1	70.1 (2)	O2^i^—La1—O1W	138.47 (12)
C9—C8—La1	167.6 (3)	O4—La1—O1W	67.64 (12)
C10—C9—C14	120.4 (4)	O2W—La1—O1W	116.19 (14)
C10—C9—C8	118.3 (4)	O3W—La1—O1W	68.43 (12)
C14—C9—C8	121.3 (4)	O1—La1—O2	69.52 (10)
C11—C10—C9	120.1 (4)	O3^i^—La1—O2	67.52 (10)
C11—C10—H10	120.0	O2^i^—La1—O2	76.19 (10)
C9—C10—H10	120.0	O4—La1—O2	47.91 (10)
C10—C11—C12	120.5 (4)	O2W—La1—O2	134.59 (11)
C10—C11—H01	119.7	O3W—La1—O2	142.73 (12)
C12—C11—H01	119.7	O1W—La1—O2	108.19 (12)
C13—C12—C11	118.9 (4)	O1—La1—Cl1	142.89 (9)
C13—C12—H02	120.5	O3^i^—La1—Cl1	76.39 (9)
C11—C12—H02	120.5	O2^i^—La1—Cl1	145.93 (8)
C12—C13—C14	122.1 (4)	O4—La1—Cl1	77.14 (9)
C12—C13—N2	118.6 (4)	O2W—La1—Cl1	73.83 (10)
C14—C13—N2	119.2 (4)	O3W—La1—Cl1	101.19 (10)
C13—C14—C9	117.9 (4)	O1W—La1—Cl1	72.00 (10)
C13—C14—H14	121.0	O2—La1—Cl1	113.19 (7)
C9—C14—H14	121.0	O1—La1—C8	71.87 (12)
C6—N1—H1A	109.5	O3^i^—La1—C8	78.12 (12)
C6—N1—H1B	109.5	O2^i^—La1—C8	99.88 (12)
H1A—N1—H1B	109.5	O4—La1—C8	23.63 (12)
C6—N1—H1C	109.5	O2W—La1—C8	149.37 (12)
H1A—N1—H1C	109.5	O3W—La1—C8	142.86 (12)
H1B—N1—H1C	109.5	O1W—La1—C8	86.58 (13)
C13—N2—H2A	109.5	O2—La1—C8	24.41 (10)
C13—N2—H2B	109.5	Cl1—La1—C8	96.26 (9)
H2A—N2—H2B	109.5	O1—La1—La1^i^	64.61 (8)
C13—N2—H2C	109.5	O3^i^—La1—La1^i^	67.42 (9)
H2A—N2—H2C	109.5	O2^i^—La1—La1^i^	41.37 (7)
H2B—N2—H2C	109.5	O4—La1—La1^i^	82.37 (8)
C1—O1—La1	138.4 (3)	O2W—La1—La1^i^	110.25 (10)
C8—O2—La1^i^	163.2 (3)	O3W—La1—La1^i^	118.34 (10)
C8—O2—La1	85.4 (3)	O1W—La1—La1^i^	130.75 (10)
La1^i^—O2—La1	103.81 (10)	O2—La1—La1^i^	34.82 (6)
La1—O1W—H11	128.0	Cl1—La1—La1^i^	139.03 (3)
La1—O1W—H21	115.2	C8—La1—La1^i^	58.74 (9)
H11—O1W—H21	116.0		

Symmetry codes: (i) −*x*+1, −*y*+1, −*z*+1.

### Hydrogen-bond geometry (Å, °)

**Table:**

*D*—H···*A*	*D*—H	H···*A*	*D*···*A*	*D*—H···*A*
N1—H1A···Cl3	0.89	2.30	3.170 (5)	167.
N1—H1B···Cl2^ii^	0.89	2.43	3.214 (5)	147.
N2—H2A···O4^iii^	0.89	2.45	3.046 (5)	125.
N2—H2A···Cl2	0.89	2.49	3.221 (4)	140.
N2—H2B···Cl3^iv^	0.89	2.28	3.169 (4)	177.
N2—H2C···Cl1^iii^	0.89	2.49	3.215 (4)	138.
N2—H2C···Cl1^v^	0.89	2.72	3.349 (5)	128.
O1W—H11···Cl2^iii^	0.81	2.39	3.186 (4)	170.
O1W—H21···Cl2^vi^	0.87	2.38	3.196 (4)	157.
O2W—H12···Cl3^vii^	0.87	2.26	3.123 (4)	172.
O2W—H22···Cl3^viii^	0.84	2.47	3.276 (4)	160.
O3W—H13···O1W	0.79	2.41	2.920 (5)	124.
O3W—H13···Cl2^vi^	0.79	2.53	3.156 (4)	137.
O3W—H23···Cl3^viii^	0.90	2.17	3.069 (4)	172.

Symmetry codes: (ii) −*x*, −*y*+1, −*z*+1; (iii) −*x*+1, −*y*+1, −*z*+2; (iv) −*x*, *y*−1/2, −*z*+1/2; (v) *x*−1, *y*, *z*; (vi) −*x*+1, *y*+1/2, −*z*+3/2; (vii) −*x*+1, *y*−1/2, −*z*+1/2; (viii) *x*+1, −*y*+3/2, *z*+1/2.
